# Prevalence of hypertension and diabetes and associated risk factors among people living with human immunodeficiency virus in Southern Ethiopia

**DOI:** 10.3389/fcvm.2023.1173440

**Published:** 2023-08-23

**Authors:** Abebe Sorsa Badacho, Ozayr Haroon Mahomed

**Affiliations:** ^1^ School of Public Health, Wolaita Sodo University, Wolaita Sodo, Ethiopia; ^2^ School of Nursing and Public Health, Public Health Medicine Discipline, University of KwaZulu-Natal, Durban, South Africa; ^3^ Health Economics and HIV and AIDS Research Division (HEARD), University of KwaZulu-Natal, Durban, South Africa; ^4^ Dasman Diabetes Institute, Kuwait City, Kuwait

**Keywords:** ART, hypertension, diabetes, non-communicable diseases, comorbidity, multimorbidity, PLWH, HIV

## Abstract

**Background:**

Access to antiretroviral therapy (ART) allows people living with HIV (PLWH) to live longer. Consequently, non communicable diseases (NCD) have emerged as the main drivers of ill health, disability, and premature death. This study assessed the magnitude of hypertension and diabetes and risk factors among PLWH receiving ART in Ethiopia.

**Methods:**

A cross-sectional study was conducted using an analytical component. Data were collected through face-to-face interviews, physical measurements, and chart reviews of the 520 adults. Associations between the demographic and clinical attributes of hypertension and diabetes were assessed using logistic regression models.

**Results:**

Prevalence of hypertension was (18.5%) (95% CI: 15.2%–21.7%), and diabetes was (6.9%) (95% CI: 4.8%–9.2%). More than two-thirds (70.8%) and 61% were newly diagnosed with hypertension and diabetes, respectively. Age > = 45 years [adjusted odds ratio (AOR) = 2.47], alcohol consumption (AOR = 4.51), Insufficient physical activity (AOR = 3.7), BMI ≥25 (AOR = 3.95), family history of hypertension (AOR = 7.1), and diabetes (AOR = 4.95) were associated with hypertension. Age ≥45 years [adjusted odds ratio (AOR) = 2.47], BMI ≥25 (AOR = 1.91), Central obesity (AOR = 3.27), detectable viral load (AOR = 4.2), hypertension (AOR = 4.95) and duration of ART >10 years (AOR = 3.12) were associated with diabetes.

**Conclusions:**

A combination of modifiable and nonmodifiable factors increased the risk of hypertension and diabetes. Primary prevention strategies, regular screening for hypertension and diabetes and integration with HIV care in primary health care are the recommended intervention measures.

## Introduction

Globally more than 38.4 million people were living with human immunodeficiency virus (HIV) in 2021 (1), an increase of more than 9 million in 2010 (1). More than 25.4 million people living with HIV (PLWH) currently use antiretroviral therapy (ART) (2). Sub-Saharan Africa remains the most severely affected by HIV, with nearly 1 in every 25 adults living with HIV, accounting for two-thirds of PLWH worldwide (2).

As a result of increased access to ART, PLWH live longer than before and have a similar life expectancy to HIV-negative persons, provided that the diagnoses are made in good time, access to medical care, and adherence to HIV treatment (3). However, comorbidity of HIV and Non-communicable diseases (NCDs) has emerged as the main driver of ill health, disability, and premature death in PLWH (4). Exposure to ART increases the risk of hyperlipidemia and diabetes; whereas some HIV medicines can increase blood glucose levels owing to the inflammatory effects of HIV (5).

More than 670,000 PLWH are in Ethiopia, and among those, 630,000 are adults over the age of 15 years. Approximately half a million (471, 721) PLWH were enrolled in ART in 2021 (1).

There is scarce evidence regarding the prevalence and risk of comorbidity of NCD with HIV in Ethiopia. A cross-sectional study conducted in Northern Ethiopia in 2018; showed that the prevalence of NCD (comorbidity of hypertension and/or diabetes) among PLWH was 19.6% (6). A study in eastern Ethiopia showed that the prevalence of hypertension and diabetes in PLWH receiving ART were 7.15% and 12.7%, respectively (7).

There is a paucity of evidence regarding the prevalence of hypertension, diabetes and other risk factors among PLWH receiving ART services from primary health care (PHC) in Ethiopia. Previous studies have reported inconsistent and inconclusive findings, mainly focusing on secondary care and referral hospital levels (6, 7). This study aimed to assess the prevalence and risk factors of hypertension and diabetes in PLWH.

## Methods

### Study design

An observational cross-sectional analytical study was conducted between January and June 2022.

### Study setting

The Wolaita zone of South Ethiopia has one teaching referral hospital, two private general hospitals, four primary hospitals, 68 health centres, and 358 health posts. Seven hospitals and seven public health centres have been providing ART follow-ups for PLWH. This study was conducted in selected PHCs that provide comprehensive HIV care services.

### Study population

The study population was PLWH aged ≥18 years enrolled in ART for at least 6 months who were being followed up for ART at five PHC facilities in South Ethiopia.

### Inclusion and exclusion criteria

Adult PLWH ≥18 years old enrolled on ART for at least 6 months, and who were being followed-up for ART in selected PHC and mentally stable who could provide informed consent were included. Patients receiving antihypertensive drugs for reasons other than hypertension and pregnant women were also excluded from the study.

### Sample size determination

The sample size was determined using Epi info and used single population proportion formula; the prevalence of NCD among PLWH was 19.6% (6), 95% of CI and α = 0.05 and desired precision *d* = 0.05 was considered. The total sample size was 532, including a design effect of 2% and 10% nonresponse rate.

### Sampling strategy

Only ten PHC facilities; of the 63 PHC facilities in the Wolaita Zone of Southern Ethiopia provide ART services. Five ART clinics from ten PHC facilities were randomly selected. The facilities were randomly selected by listing ten PHCs providing ART services, and five PHC facilities were included using the lottery method. Study participants with PLWH aged ≥18 years were identified using the ART registration book as the sampling frame. Subsequently, proportional allocation to size was made for the five PHC ART clinics. The final study participants were conveniently selected from each facility for structured interviews and measurements on a particular clinic day. Clients came to the ART clinic for HIV care and treatment.

### Data sources

Primary data were collected through structured interviews and physical measurements, and screening for hypertension and diabetes was done. In addition, secondary extraction of the clinical data from the ART registry was performed.

### Data collection

WHO stepwise tools were adapted and used in this study. Seven trained nurses working in ART clinics were recruited as the research assistants. Data were collected through structured interviews with the patients, physical measurements and screening for hypertension and diabetes. Research assistants conducted face-to-face interviews using questionnaires. Data on age, sex, marital status, educational status, occupation, monthly income, and residence were collected. Behavioural data such as alcohol consumption habits, smoking and regular physical activity were also collected.

### Measurement

Hypertension was defined as an SBP of 140 mmHg or higher, or DBP of 90 mmHg or higher, or both. The average of two BP measurements spaced 1–2 min apart was recorded. The BP was measured in both arms during the first visit to detect any possible differences. The arm with the highest value was used as the reference for subsequent measurements. The diagnosis of hypertension was confirmed by additional patient visits 1–2 weeks after the first measurement. The reported use of regular anti-hypertensive medications prescribed by professionals for increased BP was considered as hypertensive.

Random blood sugar (RBS) and fasting blood sugar (FBS) levels were measured to determine diabetes using glucometer-strip method by finger puncture. Diabetes mellitus was diagnosed if an elevated blood glucose level was recorded on two separate occasions: ≥126 mg/dl if the patient was fasting and ≥200 mg/dl if not, and those using antidiabetics were considered.

Perceived stress: was measured using the perceived stress scale (PSS), which questions feelings and thoughts during the past month. The perceived stress scale validated Cohen's (8) 10-item perceived stress scale (PSS) (9) was adapted and used. In each case, respondents were asked how often they felt a certain way on a five-point scale from “never” to “very often”. The responses to the four positively stated items were reverse-coded, and all the scale items were summed up.

Physical activity was measured according to the WHO 2020 (10) guidelines, with adults engaging in less than 150–300 min of moderate-intensity or 75–150 min of vigorous-intensity physical activity or some equivalent combination of moderate-intensity and vigorous-intensity aerobic physical activity per week (10).

The weights of the individuals were collected using a calibrated beam balance, and the scale was reset to zero before each measurement. After removing heavy clothing, the participants’ weight was measured and recorded to the closest 0.1 kg. A standard measuring scale and procedure were used to determine the height. The occiput, shoulder, buttocks, and heels all came into contact with the measuring board, and the height was measured to the closest 0.1 cm. Body mass index (BMI) is calculated as BMI = weight in kilograms/(height in m)^2^. The WHO Health Organization BIM classification was employed. The current BMI was the BMI measured during data collection. Baseline BMI was BMI taken ART initiation and extracted from the ART registration. The WHO stepwise survey tool was used to assess the dietary characteristics of fruit and vegetable consumption. Fruit and vegetable consumption was assessed by asking participants the number of days in a typical week when they eat fruits or vegetables; and when they do, the number of servings of fruit or vegetables eaten on one of those days. The WHO Health Organization recommends the consumption of fruits and vegetables more than five times per day.

### Data quality management

The research assistants were trained in the data collection and tools, sampling processes and primary ethical considerations for research.

The questionnaire was pre-tested for 5% of the sample. The pre-test was used to assess the test-retest reliability of BP and anthropometric measurements. The principal investigators communicated with supervisors daily and followed the data collection quality.

### Data analysis

Epidata 4.6.2 software and SPSS 27 software were used for the analysis of the data. Determinants of the outcome variables were identified using bivariate and multivariate logistic regression analysis. Variables with a *p*-value < 0.05 in multivariable analyses were considered statistically significant. Hosmer–Lemeshow tests assessed the model goodness-of-fit, and the normality of continuous variables was checked using a histogram.

## Result

### Socio-demographic characteristics of the study participants

Five hundred twenty (520) PLWH ≥18 years using ART services in PHC have participated.

Most of the study participants, 330 (63.5%), were females. The mean ± standard deviation age was 39. 66 ± 9.76 years, and the mean ± SD age of males was 43.33 ± 9.95, [95% CI: 41.89–44.92], and the mean age for females was 35.55 ± 9.01 [95% CI: 36.59–38.60], there was a significant difference in the mean age of male and females (*p* < 0.001).

More than two-thirds (360, 69.2%) of participants were from urban residences. Nearly a quarter of the participants had no formal education. Three hundred and sixty-two (69.6%) of the study participants had central obesity. Forty-one (7.9%) reported a family history of hypertension (Table 1).

**Table 1:** Socio-demographic characteristics of the PLWH using ART services at PHC in Wolaita, Southern Ethiopia, June 2022 (*n* = 520).

Hypertension			COR 95% CI	*P*-Value	Diabetes	COR 95% CI	*P*-Value
Age respondents	18–44	54	1		16	1	
	≥45	42	1.75 (1.12–2.77)	0.01*	20	2.73 (1.38–5.41)	0.004*
Sex of respondent	Male	35	0.99 (0.63–1.58)	0.98	19	2.04 (1.04–4.04)	0.039*
	Female	61	1		17	1	
Residence	Urban	75	1.72 (1.03–2.94)	0.04*	28	1.6 (0.71–3.6)	0.25
	Rural	21	1		8	1	
Income with a lower poverty level	Below poverty level	68	1		20	1	
	Above poverty level	28	1.88 (1.14–3.12)	0.01*	16	3.6 (1.79–7.22)	0.001**
Marriage category	Married	56	1		18	1	
	Not with spouse	40	1.21 (0.77–1.91)	0.4	18	1.7 (0.86–3.36)	0.12
Education level	No formal education	10	1		10	1	
	Formal (1–12 grades)	17	0.56 (0.33–0.93)	0.02*	17	0.59 (0.26–1.32)	0.2
	Certificate and above	9	1.88 (0.91–3.87)	0.09	9	2.97 (1.03–7.2)	0.043*
Occupation category	Employed	26	1		13	1	
	Farmer	21	0.55 (0.29–1.04)	0.07	2	0.57 (0.26–1.32)	0.181
	Housewife	34	0.86 (0.48–1.53)	0.61	14	0.39 (0.156–0.97)	0.44
	Merchant	15	0.85 (0.42–1.73)	0.65	7	0.556 (0.19–1.63)	0.28
Use tobacco products	Yes	16	2.22 (1.17–4.21)	0.01*	6	1.95 (0.77–4.94	0.16
	No	80	1		30	1	
Alcohol use	Yes	46	5.47 (3.38–8.89)	0.001**	12	2.04 (0.99–4.24)	0.054
	No	50	1		24	1	
Insufficient Physical activity	No	42	1		23	1	
	Yes	54	2.55 (1.63–4.01)	0.001**	13	0.93 (0.46–1.88)	0.84
Known family history HTN	Yes	20	5.05 (2.61–9.77)	0.001**	4	1.51 (0.51–4.5)	0.459
	No	76	1		32	1	
Known family history of DM	Yes	8	1.08 (0.48–2.42)	0.86	6	2.57 (1.001–6.58)	0.047*
	No	88	1		30	1	
Viral load	No detectable	92	1		32	1	
	Detectable	4	0.69 (0.24–2.04)	0.5	4	2.29 (0.75–6.99)	0.14
BMI current	<24.99	66	1		24	1	
	≥25	30	4.62 (2.68–7.97)	0.001**	12	3.82 (1.81–8.1)	0.001**
BMI baseline	<24.99	83	1		33	1	
	≥25	13	2.61 (1.28–5.34)	0.009**	3	1.2 (0.35–4.12)	0.77
Presence of central obesity	Yes	70	1.22 (0.74–1.97)	0.44	32	3.73 (1.29–10.74)	0.01*
	No	26	1		4	1	
DM status	No	78	1		18	1	
	Yes	18	5.21 (2.59–10.45)	0.001**	18	5.2 (2.59–10.44)	
PSS			5.05 (2.61–9.77)	0.001**		1.025 (0.95–1.09)	0.47
Duration of ART	<10 years	53	1		9	1	
	=>10 years	43	0.91 (0.58–1.42)	0.67	27	2.42 (1.18–4.95)	0.016*

* *p*-value < 0.05.

** *p*-value < 0.01.

### Prevalence of hypertension and diabetes

The prevalence of hypertension among PLWH using ART services was 18.5% (95% CI: 15.2%–21.7%). More than two-thirds (68, 70.8%) were newly diagnosed with hypertension. The overall prevalence of diabetes was 6.9% (94%, 95% CI: 4.8%–9.2%). Twenty-two (61%) with diabetes were newly diagnosed in this study.

### Comorbidity of major NCDs (hypertension or diabetes)

The overall prevalence of comorbidity of hypertension or diabetes was 21.9% (95% CI: 18.3%–25.4%) (*n* = 114).

### Prevalence of multiversity (HIV, hypertension and diabetes)

The proportion of multimorbidity of HIV, hypertension, and diabetes was 3.5% (95% CI: 1.9%–5.2%).

### Body mass index

The mean ± SD of the current BMI (measured during data collection) was 20.89 ± 3.55 kg/m^2^. The mean current BMI of males was 20.26 ± 3.19, [95% CI: 19.79–20.73], and the mean current BMI of females was 21.24 ± 3.70; [95% CI: 19.79–20.73]; the mean difference observed on current BMI between males, and females were statistically significant (*p* < 0.05). Of the study participants, 37 (7.1%) had a baseline BMI ≥25 kg/m^2^ and 68 (13.1%) had a current BMI ≥25 kg/m^2^.

The mean current BMI of hypertensive was 23.08 ± 4.05 [95% CI: 22.23–23.94] kg/m^2^ and the non-hypertensive was 20.39 ± 3.23 [95% CI: 20.11–20.71 kg/m^2^. The mean difference observed in current BMI between hypertensive, and non-hypertensive was statistically significant (*p* < 0.001).

The mean current BMI of diabetes was 21.34 ± 3.31 [95% CI: 20.33–22.50] kg/m^2^ and the non-diabetic group was 20.85 ± 3.57 [95% CI: 20.54–20.17] kg/m^2^. The mean difference in current BMI between diabetic and non-diabetic was not statistically significant (*p* > 0.05). The mean current BMI of comorbid hypertensive and diabetes patients was 24.68 ± 4.33 [95% CI: 22.87–26.83] kg/m^2^, and the mean current BMI of comorbid with hypertensive and diabetes was 20.75 ± 3.45; [95% CI: 20.43–21.05]; the mean difference observed in current BMI of comorbid hypertension or diabetes and not comorbid was statistically significant (*p* < 0.01).

The overall mean ± SD of baseline BMI (reported from the ART card) BMI measured during the first initiation of ART was 20.12 ± 3.21 kg/m^2^. The mean baseline BMI of the hypertensive was 21.60 ± 3.77 [95% CI: 20.88–22.37] kg/m^2^ and the not-hypertensive group was 19.77 ± 2.97 [19.50–20.06] kg/m^2^. The mean difference in baseline BMI between hypertensive and non-hypertensive was statistically significant (*p* < 0.001).

The mean baseline BMI of diabetic patients was 21.76 ± 3.63 [95% CI: 20.67–22.97] kg/m^2^ and the not diabetic group was 20.01 ± 3.15 [19.74–20.27] kg/m^2^ and the mean difference observed on baseline BMI between diabetic and not diabetic was statistically significant (*p* < 0.001).

The mean baseline BMI of comorbid hypertensive and diabetes patients was 23.33 ± 4.04 kg/m^2^. The mean baseline BMI of patients with comorbid hypertensive and diabetes patients was 23.33 ± 3.12 [95% CI: 21.63–25.34] kg/m^2^, and the mean baseline BMI of comorbid hypertensive and diabetes patients was 20.01 ± 4.04; [95% CI: 19.74–20.27]; the mean difference observed in baseline BMI between comorbid hypertensive or diabetes, and non-comorbid was statistically significant (*p* < 0.01).

### Behavioural characteristics

More than a third (37.7%) of the study participants had insufficient physical activity. One hundred sixty-one (31%) participants had ever consumed alcohol. One in ten (51, 9.8%) of participants have smoked tobacco products in their lifetime. More than two of the ten participants were alcohol users within 30 days of entering the study (Table 2).

**Table 2:** Behavioural characteristics of the PLWH using ART services at PHC in Wolaita, Southern Ethiopia, June 2022 (*n* = 520).

Variables	Hypertensive	*P*-Value	Diabetic	*P*-Value
Ever used any tobacco products	16 (3.1%)	0.014	6 (1.2%)	0.158
Ever consumed any alcohol	51 (9.8%)	0.001	15 (2.9%)	0.153
Consumption of any alcohol within the past 12 months	46 (8.8%)	0.001	12 (2.3%)	0.111
Current consumption of alcohol	46 (8.8%)	0.001	12 (2.3%)	0.054
Insufficient physical activity	54 (10.4%)	0.001	13 (2.5%)	0.839

### Clinical characteristics

The mean duration of ART in years was 8.81 SD = 4.2. The mean ± SD of the baseline weight of participants was 52.7 ± 9.21 rages 28–88 kg. The mean ± SD of the participant's current weight was 54.58 ± 10.02 rages 35–91 kg. The majority, 461 (88.6%) participants, had a baseline CD4 + count recorded in the ART registration, and 417 (80%) participants had recorded a recent CD4 count recent (cells/mm^3^).

### Dietary related characteristics

Approximately one in ten participants consumed fruits one to 7 times per week. None consumed five times the daily servings recommended by the WHO. Nine out of ten participants reported consuming vegetables; the weekly intake was 1–7 times a week (Table 3).

**Table 3:** Dietary-related characteristics and hypertension and diabetes status of the PLWH using ART services at PHC in Wolaita, Southern Ethiopia, June 2022.

Variables		Hypertension	*P*-value	Diabetes	*P*-value
Fruit intake category weekly	No fruit intake	15 (2.9%)	0.534	7 (1.3%)	0.298
	1–7 times per week	81 (15.6%)		29 (5.6%)	
Vegetable weekly intake category	No vegetable intake	9 (1.7%)	0.899	4 (0.8%)	0.654
	1–7 times per week	87 (16.7%)		32 (6.2%)	

### Factors associated with hypertension

Age, residence, income, education level, use of tobacco products, alcohol use, insufficient physical activity, family history of hypertension, baseline BMI, current BMI and diabetes, and Perceived Stress Scale (PSS) were candidate variables for multivariable logistic regression with *p*-value < 0.25 in bivariate analysis. After multivariable analysis, those who were insufficiently physically active were 3.7 times more likely to be hypertensive than their counterparts, AOR = 3.69 [95% CI: 2.04–6.65]. Individuals with a family history of hypertension were 7.1 times more likely to be hypertensive than their counterparts, AOR = 7.1 [95% CI: 2.87–17.54].

Individuals with a current BMI ≥25 were four times more likely to have hypertension than those with BMI <25, AOR = 3.95 [95% CI: 1.72–9.05]. Individuals with diabetes were 5 times more likely to have hypertension than their counterparts AOR = 4.95 [95% CI: 1.95–12.62]. Those who consumed alcohol were 5.3 times more likely to have hypertension than their counterparts, AOR = 5.3 [95% CI: 2.79–10.05].

The Perceived Stress Scale (PSS) was positively associated with hypertension. With each unit increase in the stress score, the risk of being hypertensive increases by 14%. In contrast, age, residence, income, education, occupation, tobacco use and baseline BMI were not significantly associated with hypertension in the final model but showed significant association in the bivariate analysis (Table 4).

**Table 4:** Final model: factors associated with hypertension among PLWH using ART services at PHC in Wolaita, Southern Ethiopia; June 2022.

		Hypertension		COR 95% CI	*P*-Value	AOR 95% CI	*P*-Value
		Yes	No				
Age respondents	18–44	54	294	1		1	
	≥45	42	130	1.75 (1.12–2.77)	0.01*	1.25 (0.71–2.21)	0.45
Income	Below poverty level	68	348	1		1	
	Above poverty level	28	76	1.88 (1.14–3.12)	0.01*	0.97 (0.45–2.10)	0.95
Residence	Urban	75	285	1.72 (1.03–2.94)	0.04*	1.5 (0.77–2.9)	0.23
	Rural	21	139	1		1	
Education category	No formal education	10	115	1		1	
	Formal (1–12 grades)	17	331	0.56 (0.33–0.93)	0.02*	2.1 (0.5–8.73)	0.31
	Certificate and above)	9	38	1.88 (0.91–3.87)	0.09	0.65 (0.35–1.18	0.15
Occupation category	Employee	26	91	1		1	
	Farmer	21	133	0.55 (0.29–1.04)	0.07	0.95 (0.34–2.65)	0.92
	Housewives	34	138	0.86 (0.48–1.53)	0.61	2.58 (0.97–6.9)	0.06
	Merchants	15	62	0.85 (0.42–1.73)	0.65	2.17 (0.74–6.37)	0.16
Use tobacco products	Yes	16	35	2.22 (1.17–4.21)	0.01*	2.26 (0.92–5.54)	0.07
	No	80	389	1		1	
Insufficient Physically active	No	42	282	1		1	
	Yes	54	142	2.55 (1.63–4.01)	0.001**	3.69 (2.04–6.65)	0.001**
Known family HTN	Yes	20	21	5.05 (2.61–9.77)	0.001**	7.1 (2.87–17.54)	0.001**
	No	76	403	1		1	
BMI current category	<24.99	66	386	1		1	
	≥25	30	38	4.62 (2.68–7.97)	0.001**	3.95 (1.72–9.05)	0.001**
Alcohol use	Yes	46	61	5.47 (3.38–8.89)	0.001**	5.3 (2.79–10.05)	0.001**
	No	50	363	1		1	
BMI baseline	<24.99	83	400	1	1	1	
	≥25	13	24	2.61 (1.28–5.34)	0.009*	1.2 (0.42–3.42)	0.73
DM status	No	78	406	1		1	
	Yes	18	18	5.21 (2.59–10.45)	0.001**	4.95 (1.95–12.62)	0.001**
PSS	Mean = 20.74; SD = 4.93			1.12 (1.06–1.17)	0.001**	1.14 (1.08–1.22)	0.001**

* *p*-value < 0.05.

** *p*-value < 0.01.

### Factors associated with diabetes

In the multivariable logistic regression analyses, Individuals with age ≥45 years AOR = 2.47 [95% CI: 1.12–5.45]; Individuals with central obesity AOR = 3.27 [95% CI: 1.07–9.98]; Individuals with detectable viral load AOR = 4.2 [95% CI: 1.18–15.4]; hypertension AOR = 4.68 [95% CI: 1.94−11.35]; ART duration greater than 10 years AOR = 3.12 [95% CI: 1.33–7.33]; current BMI ≥25 AOR = 4.95 [95% CI: 1.95–12.62] were significantly associated with diabetes. In contrast, in multivariable analysis, sex, income level, and family history of diabetes were not significantly associated with diabetes (Table 5).

**Table 5:** Final model: factors associated with diabetes among PLWH using ART services at PHC in Wolaita, Southern Ethiopia; June 2022.

		Diabetes		COR 95% CI	*P*-Value	AOR 95% CI	*P*-Value
		Yes	No				
Age	18–44	16	332	1		1	
	≥45	20	152	2.73 (1.38–5.41)	0.004**	2.47 (1.12–5.45)	0.02*
Sex	Male	19	171	2.04 (1.04–4.04)	0.039*	1.4 (0.6–3.27)	0.43
	Female	17	313	1		1	
Income level	Below poverty line	20	396	1		1	
	Above poverty line	16	88	3.6 (1.79–7.22)	0.001**	2.22 (0.69–7.07)	0.17
Alcohol use	Yes	12	95	2.04 (0.99–4.24)	0.054	0.77 (0.3–1.97)	0.59
	No	24	389	1			
Known family history of DM	Yes	6	35	2.57 (1.001–6.58)	0.049*	2.22 (0.69–7.06)	0.17
	No	30	449	1		1	
Viral load	Not detectable	32	459	1		1	
	Detectable	4	25	2.29 (0.75–6.99)	0.14	4.23 (1.18–15.4)	0.02*
Recent BMI	<24.99	24	428	1		1	
	≥25	12	56	3.82 (1.81–8.1)	0.001**	1.91 (1.21–4.8)	0.04*
Presence of central obesity	Yes	32	330	3.73 (1.29–10.74)	0.01*	3.27 (1.07–9.98)	0.03*
	No	4	154	1			
Hypertension status	No	18	406	1		1	
	Yes	18	78	5.2 (2.59–10.44)		4.68 (1.94–11.35)	0.001*
Duration of ART	<10 years	9	265	1		1	
	=>10 years	27	219	2.42 (1.18–4.95)	0.016*	3.12 (1.33–7.33)	0.01*

* *p*-value < 0.05.

** *p*-value < 0.01.

## Discussion

This study aimed to determine the prevalence of hypertension and diabetes and identify its associated factors among PLWH undergoing ART from PHC facilities. The overall prevalence of hypertension was 18.5%.

This study finding reported a higher prevalence of hypertension than some studies conducted in Sub-Saharan African countries, 10.2% in Zimbabwe (11) and 12.4% in Kenya (12) and previous studies in Ethiopia, 14.1%, the Bahir Dar North Ethiopia (6), 12.7% Harar Eastern Ethiopia (7) and 14.0% in Debremarkos Northwest Ethiopia (13).

In this study, we assessed the prevalence of hypertension and associated factors in PLWH who received ART from PHC settings where no routine hypertension screening was done. Most of these patients remain unaware of their hypertension status. Previous studies reported similar (14) in Ethiopia; PLWH care focus is limited to HIV care, and NCD have less attention (15). This indicates the need for regular screening for hypertension and diabetes for PLWH during ART services provision, and ART clinics could not only focus on HIV care but the need for hypertension and diabetes care.

More than two-thirds (68, 70.8%) were newly diagnosed with hypertension and were not aware of their hypertension status. The study finding was consistent with a study conducted at Dessie, Northeast Ethiopia, that 74.4% of PLWH screened for hypertension were newly diagnosed (16); in the western Brazilian Amazon, only one-third had prior knowledge of their hypertension status (17). Lack of regular screening for hypertension unknowingly causes harm to the health of the patients and hinders the aim of ART services.

In our study, more than two-thirds of PLWH (362, 69.6%) never measured their blood pressure to know their hypertension status before this study. Our study finding was higher than findings from a study conducted in western Brazilian 44.3% of participants reported that blood pressure was never measured (17).

This finding implies a lack of routine hypertension screening in Ethiopia at the PHC level for PLWH despite the increasing number of hypertension cases among PLWH. This calls for health systems to pay attention to routine screening at the PHC level.

The prevalence of diabetes was (6.9%) and 61% with diabetes newly diagnosed in this study.

Our findings showed a higher prevalence of diabetes among PLWH compared to in Hosanna general hospitals of Ethiopia, 5.7% (18), and in the study conducted in Zambia, 5% (19).

### Risk factors associated with developing hypertension among PLWH

In this study, significant modifiable factors such as alcohol consumption, insufficient physical activity, current BMI, perceived stress level, and nonmodifiable factors (family history of hypertension and diabetes status) were found to be significantly associated with hypertension among PLWH receiving ART.

Those who consumed alcohol were 5.3 times more likely to be hypertensive than their counterparts. This finding was consistent with other studies that alcohol use was associated with hypertension in the PLWH study of Harar in eastern Ethiopia (7) and Butajira Southern Ethiopia (20). A systematic review and meta-analysis showed that alcohol intake increases the risk of hypertension, and limiting alcohol intake should be advised for both men and women (21). Previous studies reported the modest protective effect of alcohol consumption in women at low doses (21, 22).

Those who were overweight or obese were a risk of developing hypertension. Individuals with a current BMI ≥25 were 4 times more likely to be hypertensive than compared with BMI <25. This finding is in line with other studies (6, 7, 17, 23–26) that found high BMI was an associated risk factor for hypertension among PLWH.

This study was consistent with studies in Ethiopia. The study of Bahir Dar, Northern Ethiopia, reported that an increased BMI was associated with NCD comorbidity; individuals with BMI >25 were 2.7 times more develop NCD comorbidity (6). Debre Markos, Northwest Ethiopia, reported that BMI was associated with hypertension, with BMI >25 being 3.32 times higher developing hypertension (27). Also, a study conducted in Dessie, Northeast Ethiopia, reported that a higher BMI was significantly associated with hypertension, with BMI >25 being 2.87 times higher developing hypertension than a normal BMI (16).

A possible reason might be the gradient of increasing blood pressure with higher BMI levels. BMI measurement taken during ART follow-up can be used as a simple and effective predictor of hypertension among PLWH. Counselling to improve eating habits and increase physical activity is vital in controlling BMI.

Physical inactivity is a modifiable risk factor for hypertension. Those who were insufficiently physically active were 3.7 times more likely to be hypertensive than their counterparts. This finding was consistent with Debre Markos, Northwest Ethiopia participants who did not exercise regularly were 2.72 times higher risk of hypertension than those who did physical exercise (27). The possible reason might be that low physical activity levels are directly linked to weight gain. It requires attention during counselling for regular physical activities.

The Perceived Stress Scale (PSS) was positively associated with hypertension in this study. With each unit increase in the stress score, the risk of being hypertensive increases by 14%. Stress may not directly cause hypertension but can lead to repeated blood pressure elevations, which may lead to hypertension. In this study, stress was prevalent among PLWH and associated with the development of hypertension.

In our study, a family history of hypertension was significantly associated with hypertension. Individuals with a family history of hypertension were 7.1 times more likely to be hypertensive than their counterparts. Knowing family history is an essential nonmodifiable risk factor for hypertension. Studies have found that the prevalence of hypertension is significantly higher in those with a family history of hypertension than in those with no family history (28, 29). Our study finding was consistent with a study conducted in Ghana among PLWH reported that a family history of hypertension was associated with hypertension; individuals with a family history of hypertension were 1.43 times higher developing hypertension (28). A study from South Ethiopia reported that a family history of hypertension was associated with hypertension among the general population; individuals with a family history of hypertension were 2.57 times higher with no family history of hypertension (29). A study in Sri Lanka reported that adults with a family history of hypertension had a 1.4 times higher risk of developing hypertension (30). The Sri Lanka study was a population-based survey, and our study focused on PLWH-received ART.

In our study, diabetes was significantly associated with hypertension among PLWH. Individuals with diabetes were five times more likely to be hypertensive than their counterparts. This finding is similar to other studies from Butajira Southern Ethiopia that reported individuals having comorbidity of diabetes were 5.29 times more likely to develop hypertension (20). Moreover, a study at Dessie Referral Hospital in Northeast Ethiopia reported that individuals with diabetes were 2.76 times more likely to develop hypertension (16).

### Factors associated with diabetes among PLWH

Age, central obesity, viral load, hypertension status, current BMI and ART duration in years were significantly associated with diabetes among PLWH using ART.

Individuals with central obesity in PLWH were 3.3 times more likely to develop diabetes than their counterparts, consistent with Cameroon (25). Increased abdominal circumference due to central body fat accumulation and excess weight is associated with an increased risk of diabetes, mainly due to metabolic and cardiovascular changes (19, 31, 32). PLWH needs to focus not only on ART but also on managing chronic comorbidities.

Individuals aged over 45 years were 2.5 times more likely to develop diabetes than those under 45 years. This finding was in line with other studies that found that older age was significantly associated with developing diabetes.

Also, the duration of ART was significantly associated with diabetes; staying more than ten years at ART was 3 times more likely to develop diabetes compared to ART duration of less than 10 years. It was consistent with other studies; A study in Ethiopia Bahir Dar (6), Jimma referral hospital (33) and Jimma Zone hospitals (34).

Individuals with detectable viral loads were four times more likely to be diabetic in the PLWH. The detectable plasma viral load was significantly associated with diabetes in PLWH patients. Poor control of viral load is directly correlated with poor diabetes control (35). Poor adherence to ART correlates with poor adherence to treatment for other medical comorbidities, explaining the relationship between poor management of both conditions (35).

Individuals with hypertension were 4.7 times more likely to develop diabetes than their counterparts. This study's findings were consistent with those elsewhere (7, 33).

Patients with high blood pressure (hypertension) are at a greater risk of developing diabetes than those with normal blood pressure. A possible reason is that patients with hypertension often exhibit insulin resistance and are more prone to developing diabetes. Hypertension is a significant risk factor for diabetes associated with vascular complications and characterised by vascular dysfunction and injury (36). A current BMI ≥25 was significantly associated with the development of diabetes. Other Previous studies were conducted in Ethiopia, Jimma Zone hospital (34), and Northwest Ethiopia hospitals (37), Bahir Dar (6). Obese and overweight states are common in PLWH, and an increased risk of incident diabetes has been noted with weight gain after ART initiation in PLWH (38). The higher incidence of hypertension was significantly associated with high BMI among PLWH (39). An increased in BMI leads to an increased risk for hypertension (40, 41). The possible association of current BMI could be that ART drugs correlate with rapid weight gain during ART drug use (42). It indicates that the need to focus on risk reduction strategies for overweight and obese individuals should be the primary focus.

### Limitations of the study

In this study, there may have been selection and social desirability biases. Selection bias might have been introduced in our research because PLWH receiving ART services from primary health care participants underestimated the prevalence of hypertension and diabetes among PLWH, which might represent all PLWH. Social desirability bias might be introduced when assessing risk factors like alcohol consumption and smoking behaviour in PLWH, as the data collectors were nurses in an ART clinic providing routine ART care in the same clinic. Also, the study design being a cross-sectional one should be noted as a limitation.

## Conclusions and recommendations

There is an increased prevalence of hypertension and diabetes among PLHWs using ART from primary healthcare facilities in southern Ethiopia. Most participants were unaware of their hypertension and diabetes status before this study. Combinations of modifiable and nonmodifiable factors increase the risk of both these diseases. Primary prevention strategies (health promotion), regular screening for hypertension and diabetes and integration with HIV care in primary health are recommended as immediate intervention measures.

## Data Availability

The original contributions presented in the study are included in the article/Supplementary Material, further inquiries can be directed to the corresponding author.
